# Development and validation of an explainable machine learning model using routine laboratory biomarkers for identifying prevalent MASLD: Evidence from two observational studies

**DOI:** 10.1007/s10238-026-02163-x

**Published:** 2026-05-15

**Authors:** Jialin Wu, Wenting Wei, Terry Cheuk-Fung Yip, Xinyi Deng, Bonan Chen, Yang Lyu, Peiyao Yu, Tiejun Feng, Fuda Xie, Ge Zhang, Kangmin Zhuang, Aimin Li, Wei Kang

**Affiliations:** 1 Department of Anatomical and Cellular Pathology, State Key Laboratory of Translational Oncology, Prince of Wales Hospital, The Chinese University of Hong Kong, Sir Y.K. Pao Cancer Center, Hong Kong, China; 2 Institute of Digestive Disease, State Key Laboratory of Digestive Disease, Li Ka Shing Institute of Health Science, The Chinese University of Hong Kong, Hong Kong, China; 3 CUHK-Shenzhen Research Institute, Shenzhen, China; 4 Zhongshan School of Medicine, Sun Yat-Sen University, Guangzhou, China; 5 Medical Data Analytics Centre, Department of Medicine and Therapeutics, The Chinese University of Hong Kong, Hong Kong, China; 6 Department of Endocrinology, The First Affiliated Hospital, Sun Yat-Sen University, Guangzhou, China; 7 Law Sau Fai Institute for Advancing Translational Medicine in Bone and Joint Diseases (TMBJ), School of Chinese Medicine, Hong Kong Baptist University, Hong Kong, China; 8 Guangdong Provincial Key Laboratory of Gastroenterology, Department of Gastroenterology, Nanfang Hospital, Southern Medical University, Guangzhou, China

**Keywords:** Machine learning, Metabolic dysfunction-associated steatotic liver disease, Plasma biomarker, Screening model

## Abstract

Although many predictive models for metabolic dysfunction-associated steatotic liver disease (MASLD) have been developed, their performance remains suboptimal. We aimed to develop an interpretable machine learning (ML)-based plasma biomarker model for identifying prevalent MASLD. Data from the National Health and Nutrition Examination Survey (NHANES 2017–2020) were randomly divided into a training cohort (N = 2760) and an internal cohort (N = 1184). Eleven ML algorithms were employed to construct classification models. Model interpretability was visualized via the SHapley Additive exPlanations (SHAP) method. External validation of these models was further conducted using data from the Korea NHANES (KNHANES) 2019–2021. The association between the selected features and prevalent MASLD was evaluated using restricted cubic spline regression analysis. Feature selection was performed using LASSO regression and the Boruta algorithm. Key predictors included diabetes mellitus (DM), waist circumference (WC), age, hypertension, and atherogenic index of plasma (AIP). All evaluated ML algorithms demonstrated robust predictive capabilities, with areas under the curve (AUC) exceeding 0.70. Among these, the Extra Trees (ET) performed the best, achieving an AUC of 0.879 (95% CI 0.856–0.897) in the internal testing group and maintaining good performance in the external KNHANES cohort with an AUC of 0.822 (95% CI 0.815–0.829). The DeLong test revealed significant differences in AUC between ET and other algorithms. These findings suggest that age, WC, DM, hypertension, and AIP are informative features associated with prevalent MASLD. The ET model showed strong discriminative performance and may serve as a practical tool for MASLD screening.

**Graphical Abstract:**

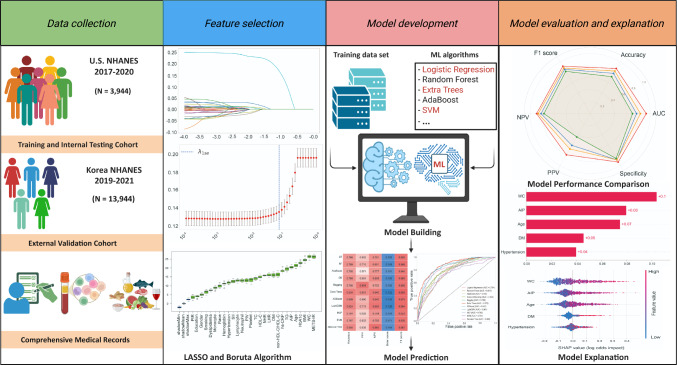

**Supplementary Information:**

The online version contains supplementary material available at 10.1007/s10238-026-02163-x.

## Introduction

Metabolic dysfunction-associated steatotic liver disease (MASLD) is a leading cause of chronic liver disease [1], with a global prevalence of 38% among adults [2]. MASLD is characterized by excessive fat deposition within hepatocytes, not only causing severe liver conditions such as liver fibrosis, cirrhosis and liver cancer [3, 4], but also increasing patients’ risk of cardiovascular diseases [5], chronic kidney disease [6], type 2 diabetes, metabolic syndrome, insulin resistance (IR) [7] and extrahepatic cancers [8]. These conditions have a negative impact on patients’ quality of life and impose a great burden on global healthcare. Therefore, it is crucial to develop an effective, accessible, and robust model for screening and identifying prevalent MASLD in routine clinical practice.

The pathophysiology of MASLD involves excess energy delivery, adipocyte dysfunction, IR and de novo lipogenesis. These procedures result in free fatty acid release and toxic lipid species production, induce the injury and death of hepatocyte, and finally lead to chronic inflammation and fibrogenesis [9]. Emerging studies have explored many clinical biomarkers for identifying MASLD, among them, lipid profile indices may have predictive potential for the diagnosis and prognosis of MASLD and its complications [10–12]. Recent studies have demonstrated the predictive performance of the plasma atherogenicity index (AIP) and non-high-density lipoprotein cholesterol/high-density lipoprotein cholesterol (non-HDL-C/HDL-C) ratio, emphasized their validity compared to other common lipid markers like total cholesterol (TC), and low-density lipoprotein cholesterol (LDL-C) [13, 14].

Furthermore, the molecular mechanisms driving MASLD involve dysregulated lipid metabolism, chronic inflammation, oxidative stress, and immune response [15]. Inflammation participated in the pathogenesis of MASLD by impairing liver parenchymal cell [16], causing mitochondrial dysfunction [17] and affecting intestinal flora [18]. In addition, changes in liver immune cell composition during non-alcoholic steatohepatitis lead to parenchymal cell damage and dysfunction [19]. Studies found that immuno-inflammatory biomarkers, like lymphocyte-to-high-density lipoprotein ratio (LHR) [20], neutrophil-to-high-density lipoprotein cholesterol ratio (NHR) [21], systemic immune inflammation index (SII) [22], and pan-immune-inflammation value (PIV) [23] also have potential to detect high-risk MASLD individuals.

Machine learning (ML) has the ability to handle large, complex and irrelevant datasets or indices [24], offering a comprehensive and accurate amalgam that traditional analytic methods often lack. A recent study reported that ML models using demographic and baseline varieties can effectively screen for MASLD in the general population [25]. However, existing models mainly focus on demographic and clinical features or combine these with single immuno-inflammatory biomarkers, and their discriminatory performance remains suboptimal. Here, we aim to develop and validate an interpretable ML model for identifying prevalent MASLD by comprehensively integrating demographic characteristics and plasma biomarkers, including metabolic and immuno-inflammatory indices.

## Materials and methods

### Study design and participants

This study analyzed the data from using the U.S. National Health and Nutrition Examination Surveys (NHANES 2017–2020) and Korean NHANES (KNHANES 2019–2021). The U.S. NHANES and Korean NHANES represent a comprehensive, continual cross-sectional study that systematically collects a diverse array of data aimed at exploring various health-related issues.

**Fig. 1:**
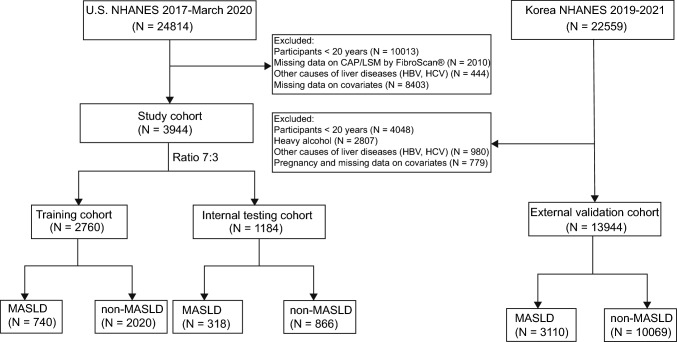
Flow chart of the study cohort selection. NHANES, National Health and Nutrition Examination Survey; MASLD, metabolic dysfunction-associated steatotic liver disease; CAP, controlled attenuation parameter; LSM, liver stiffness measurement

In our study, we analyzed data from NHANES conducted between 2017 and 2020, encompassing a total of 24,814 subjects. We employed the controlled attenuation parameter (CAP) for the diagnosis of MASLD. Participant selection followed a systematic process as illustrated in Fig. 1. Our exclusion criteria included: (1) age < 20 years; (2) missing complete components for CAP or liver stiffness measurement (LSM); (3) excessive alcohol consumption (alcohol consumption > 20 g/day for male and 10 g/day for female) [26]; and (4) other pre-existing liver conditions, including viral hepatitis infection (positive HCV RNA, HCV antibody or HBsAg test). Participants with incomplete data were excluded from the analysis, and no imputation was performed to ensure robustness in our analysis. Consequently, a final cohort of 3944 participants were included. All data underwent rigorous cleaning and processing to facilitate the development of ML models. The work has been reported in line with the STROCSS criteria [27].

### Definition of MASLD

The evaluation of liver steatosis and fibrosis was conducted using the FibroScan® model 502 V2 Touch (Echosens, Paris, France) to conduct the elastography exam in the NHANES Mobile Examination Center, and the values of median CAP and LSM were calculated by the device along with the interquartile range [28, 29]. Complete data were defined as fasting time of at least 3 h, 10 or more complete stiffness measures, and a liver stiffness interquartile range/median < 30%. In this study, Participants with CAP ≥ 280 dB/m were diagnosed with steatotic liver disease (SLD) [30]. MASLD is defined as SLD in addition to at least one of the five cardiometabolic criteria without viral hepatitis or significant alcohol consumption [31]: (1) body mass index (BMI) of ≥ 25 kg/m [2] or waist circumference (WC) of ≥ 94 cm for men or ≥ 80 cm for women; (2) fasting blood glucose levels of ≥ 100 mg/dL or glycosylated hemoglobin levels of ≥ 5.7%, a history of type 2 diabetes, or currently receiving treatment for type 2 diabetes; (3) blood pressure readings of ≥ 130/85 mmHg or undergoing antihypertensive treatment; (4) plasma triglyceride levels of ≥ 150 mg/dL or currently receiving lipid-lowering therapy; (5) levels of HDL-C of < 40 mg/dL for men or < 50 mg/dL for women, or the use of lipid-lowering medication.

Conventional hepatic steatosis indices include fatty liver index (FLI), hepatic steatosis index (HSI) score and Framingham steatosis index** (**FSI), which are calculated as follows [32–34]:

$$\text{FLI }=\frac{({{\boldsymbol{e}}}^{0.953\times \mathbf{ln}\left({\boldsymbol{T}}{\boldsymbol{G}}\right)+0.139\times {\boldsymbol{B}}{\boldsymbol{M}}{\boldsymbol{I}}+0.718\times \mathbf{ln}\left({\boldsymbol{G}}{\boldsymbol{G}}{\boldsymbol{T}}\right)+0.053\times {\boldsymbol{W}}{\boldsymbol{C}}-15.745})}{(1+{{\boldsymbol{e}}}^{0.953\times \mathbf{ln}\left({\boldsymbol{T}}{\boldsymbol{G}}\right)+0.139\times {\boldsymbol{B}}{\boldsymbol{M}}{\boldsymbol{I}}+0.718\times \mathbf{ln}\left({\boldsymbol{G}}{\boldsymbol{G}}{\boldsymbol{T}}\right)+0.053\times {\boldsymbol{W}}{\boldsymbol{C}}-15.745})}\times 100$$

$$\text{HSI score}=8\times \mathbf{A}\mathbf{L}\mathbf{T}/\mathbf{A}\mathbf{S}\mathbf{T}(\mathbf{U}/\mathbf{L})+\mathbf{B}\mathbf{M}\mathbf{I}(\mathbf{k}\mathbf{g}/\mathbf{m}^2)(+2,\mathbf{i}\mathbf{f}\,\mathbf{d}\mathbf{i}\mathbf{a}\mathbf{b}\mathbf{e}\mathbf{t}\mathbf{e}\mathbf{s}),(+2,\mathbf{i}\mathbf{f}\,\mathbf{w}\mathbf{o}\mathbf{m}\mathbf{e}\mathbf{n})$$

$$\begin{aligned} {\mathrm{FSI}} & = - 7.981 + 0.011 \times {\mathbf{age}}\,({\mathbf{years}}) - 0.146 \times {\mathbf{sex}}\,({\mathbf{female}} = 1,{\mathbf{male}} = 0) \\ & + 0.173 \times {\mathbf{BMI}}\,({\mathbf{kg}}/{\mathbf{m}}^{2} ) + 0.007 \times {\mathbf{triglycerides}}\,({\mathbf{mg}}/{\mathbf{dL}}) \\ & + 0.593 \times {\mathbf{hypertension}}\,({\mathbf{yes}} = 1,{\mathbf{no}} = 0) + 0.789 \times {\mathbf{diabetes}}\,({\mathbf{yes}} = 1,{\mathbf{no}} = 0) \\ & + 1.1 \times {\mathbf{ALT}}/{\mathbf{AST}}\,{\mathbf{ratio}} \ge 1.33({\mathbf{yes}} = 1,{\mathbf{no}} = 0) \\ \end{aligned}$$

BMI represents body mass index; AST represents aspartate aminotransferase; ALT represents alanine aminotransferase.

### Assessment of plasma indices

The formulas of indices are shown below:

non-HDL-C/HDL-C = (TC—HDL-C)/HDL-C [13];

NHR = neutrophil count (${10}^{3}$ cells/μL)/HDL-C(mmol/L) [21];

LHR = lymphocyte count (${10}^{3}$ cells/μL)/HDL-C(mmol/L) [20];

SII = (peripheral platelet count × neutrophil absolute value) / lymphocyte absolute.

value [22];

PIV = (platelet count × neutrophil count × monocyte count)/ lymphocyte count [23];

AIP = ${log}_{10}$(TG (mg/dL)/HDL-C(mg/dL)) [14];

### Data preprocessing and feature selection

We employed several advanced ML methods to examine the covariance among variables and their subsequent impact on our analytical outcomes. To enhance the interpretability of the ML models, we performed a feature selection process to identify candidate variables most strongly associated with prevalent MASLD status. The least absolute shrinkage and selection operator (LASSO) regression and the Boruta algorithm were utilized for identifying informative features. The findings from the two-step feature selection method were synthesized, allowing us to isolate variables that consistently demonstrated significance across approaches for the development of the foundational predictive model. The final basic model variables included were age, WC, diabetes mellitus (DM), hypertension, and AIP.

### Construction of the ML model

The dataset was randomly divided into a training set (70%) and a testing set (30%). We applied eleven distinct machine learning models, including logistic regression (LR), decision trees (DT), extremely randomized trees (ET), random forest (RF), bootstrap aggregating (Bagging), adaptive boosting (AdaBoost), gradient boosting (GBoost), extreme gradient boosting (XGBoost), light gradient boosting machine (LightGBM), multilayer perceptron (MLP), and support vector machine (SVM), to investigate the association between MASLD and the selected variables. Each model was optimized using its specific feature subset derived from the training dataset, with performance evaluated on the independent testing set.

After aggregating the discriminative features from each model, we selected the most effective approach for disease identification. Additionally, we utilized SHapley Additive exPlanations (SHAP) values to provide interpretability to the models.

### Evaluation of ML model

The main metric utilized was the area under the curve (AUC). Higher AUC values indicate superior discriminatory power. Alongside AUC, several additional metrics were assessed, including accuracy, sensitivity, and specificity, positive predictive value (PPV), negative predictive value (NPV), Brier score and F1 score were also included. DeLong test was employed to evaluate the disparities in the AUC between different models.

### External validation using KNHANES data

External validation using the cross-sectional data from the 2019–2021 KNHANES [35]. Because KNHANES does not provide transient elastography measurements, hepatic steatosis was defined using HSI ≥ 36. The KNHANES datasets are publicly available on the KNHANES website (http://knhanes.cdc.go.kr). More details of KNHANES and related ethnic statement are described in Supplementary method.

Individuals with MASLD were classified as lean and non-lean MASLD based on BMI. Lean MASDL was defined as BMI < 25 kg/m^2^ NHANES and ≥ 23 kg/m^2^ in KNHANES.

### Statistical analysis

All analyses were performed by R (version 4.4.1) and Python (Version 3.12.7). In this study, continuous variables were reported as mean ± standard deviation (SD) for those following a normal distribution and homogeneity of variance, while non-normally distributed data were expressed as median with interquartile range. Categorical variables were summarized as percentages and frequencies. Characteristics were compared using the t-test for normally distributed continuous variables, Mann–Whitney U test for skewed distributions, and the weighted chi-squared test for categorical variables. *P* < 0.05 was considered statistically significant.

### Ethics approval and consent to participate

The research adhered to the principles of the Declaration of Helsinki. Informed consent was not required owing to the retrospective nature of the study.

## Results

### Baseline characteristics

This study finally included 3944 subjects and divided into two distinct cohorts: a training cohort consisting of 2760 individuals and an internal testing cohort comprising 1184 participants. Comprehensive descriptions of demographic characteristics, medical history, laboratory assessments, and clinical indices were summarized in Table 1. The prevalence of MASLD in training and internal testing cohort were 26.8% and 26.9%, respectively. All baseline variables showed no significant difference between training cohort and internal testing cohort (*P* > 0.05).

**Table 1:** Characteristics and outcomes of the study population

Characteristics	Training cohort	Internal testing cohort	*P*-value
	(N = 2760)	(N = 1184)	
Demographic characteristics			
Age	51.0 (35.00, 64.00)	51.0 (36.00, 63.00)	0.468
Gender, n (%)			0.560
Female	1432 (51.9%)	627 (53.0%)	
Male	1328 (48.1%)	557 (47.0%)	
Race, n (%)			0.796
Mexican American	359 (13.0%)	163 (13.8%)	
Non-Hispanic White	994 (36.0%)	426 (36.0%)	
Non-Hispanic Black	655 (23.7%)	293 (24.7%)	
Other Hispanic	271 (9.8%)	110 (9.3%)	
Other races	481 (17.4%)	192 (16.2%)	
PIR, n (%)			0.354
< 1	475 (17.2%)	219 (18.5%)	
≥ 1	2285 (82.8%)	965 (81.5%)	
Education level, n (%)			0.727
Less than high school	497 (18.0%)	207 (17.5%)	
High school graduate +	2263 (82.0%)	977 (82.5%)	
BMI (kg/)	29.0 (24.80, 34.30)	28.7 (24.78, 33.82)	0.697
Waist circumference(cm)	100.0 (88.60, 112.70)	99.3 (88.30, 111.78)	0.281
Smoking, n (%)			0.869
No	1620 (58.7%)	699 (59.0%)	
Yes	1140 (41.3%)	485 (41.0%)	
Metabolic syndrome			
DM, n (%)			0.136
No	2082 (75.4%)	920 (77.7%)	
Yes	678 (24.6%)	264 (22.3%)	
Hypertension, n (%)			0.869
No	1522 (55.1%)	657 (55.5%)	
Yes	1238 (44.9%)	527 (44.5%)	
Dyslipidemia, n (%)			0.990
No	809 (29.3%)	348 (29.4%)	
Yes	1951 (70.7%)	836 (70.6%)	
METS-IR	43.3 (35.27, 52.98)	43.0 (34.95, 52.19)	0.309
Laboratory test			
Lymphocyte (/L)	2.0 (1.60, 2.40)	2.0 (1.60, 2.40)	0.751
Monocyte (/L)	0.5 (0.40, 0.60)	0.5 (0.40, 0.60)	0.087
Neutrophil (/L)	3.7 (2.90, 4.70)	3.7 (2.88, 4.80)	0.503
Platelet (/L)	234.0 (197.00, 275.00)	236.0 (196.00, 278.00)	0.457
Hemoglobin (g/dL)	14.1 (13.20, 15.20)	14.1 (13.10, 15.10)	0.089
HbA1c (%)	5.6 (5.30, 6.00)	5.6 (5.30, 6.00)	0.865
HDL-C (mg/dL)	50.0 (41.00, 60.00)	51.0 (42.00, 60.00)	0.079
hs-CRP (mg/L)	2.0 (0.89, 4.45)	1.8 (0.81, 4.29)	0.110
TG (mg/dL)	92.0 (62.0, 138.0)	91.0 (59.0, 131.0)	0.237
TC (mg/dL)	180.0 (156.00, 208.00)	181.0 (156.00, 210.00)	0.682
Indices			
NHR	2.8 (1.97, 3.98)	2.8 (1.90, 4.07)	0.853
LHR	1.5 (1.12, 2.05)	1.5 (1.11, 2.06)	0.566
AIP	-0.10 (-0.32, 0.13)	-0.10 (-.33, 0.11)	0.138
non-HDL-C/HDL-C ratio	2.6 (1.87, 3.43)	2.5 (1.80, 3.37)	0.312
SII	434.9 (309.92, 618.49)	439.6 (308.89, 622.56)	0.610
PIV	220.1 (141.55, 345.83)	223.6 (144.07, 367.87)	0.385
CAP (dB/m)	266.0 (219.75, 314.00)	264.0 (220.00,311.00)	0.596
LSM (kPa)	5.0 (4.10, 6.20)	5.1 (4.10, 6.20)	0.918
HSI	38.5 (32.96, 44.79)	38.0 (33.00, 44.69)	0.343
FLI	56.9 (21.40, 86.59)	53.2 (20.11, 85.28)	0.236
FSI	-1.2 (-2.31, 0.06)	-1.3 (-2.38, -0.10)	0.286
Outcome measurements			
MASLD, n (%)			1.000
No	2020 (73.2%)	866 (73.1%)	
Yes	740 (26.8%)	318 (26.9%)	

Note: Continuous variables are presented as median (IQR). Categorical variables are presented as n (%)

Abbreviations: PIR, poverty income ratio; BMI, body mass index; DM, diabetes mellitus; METS-IR, metabolic score for insulin resistance; HDL-C, high-density lipoprotein cholesterol; hs-CRP, hypersensitive-c-reactive-protein; TG, triglycerides; TC, total cholesterol; NHR, neutrophil-to-high-density lipoprotein cholesterol ratio; LHR, lymphocyte-to-high-density lipoprotein ratio; AIP, atherogenic index of plasma; non-HDL-C/HDL-C, non-high-density lipoprotein cholesterol to high-density lipoprotein cholesterol ratio; SII, systemic immune inflammation index; PIV, pan-immune-inflammation value; CAP, controlled attenuation parameter; LSM, liver stiffness measurement; FLI, fatty liver index; FSI, Framingham steatosis index; HIS, hepatic steatosis index; MASLD, metabolic dysfunction associated steatotic liver disease

Table S1 presented the comparison of baseline variables between MASLD and non-MASLD groups. Among demographic characteristics, the median age of MASLD patients were higher than the non-MASLD (*P* < 0.001). The MASLD group also had higher BMI and WC in comparison with the non-MASLD group (*P* < 0.001). As to metabolic syndrome, participants who were diagnosed with MASLD were more likely to suffer from DM, hypertension and dyslipidemia compared with non-MASLD (*P* < 0.001). Laboratory test demonstrated that MASLD individuals displayed increased levels of neutrophil, HbA1c and hypersensitive-c-reactive-protein than those without MASLD (all *P* < 0.001). Distinctively, the HDL cholesterol level was lower in the MASLD group than the non-MASLD group (*P* < 0.001). All the metabolic and immuno-inflammatory indices showed significant differences between these two groups (all *P* < 0.001). Specifically, the MASLD group had significantly higher CAP and LSM value than non-MASLD populations.

### Feature selection in ML

**Fig. 2:**
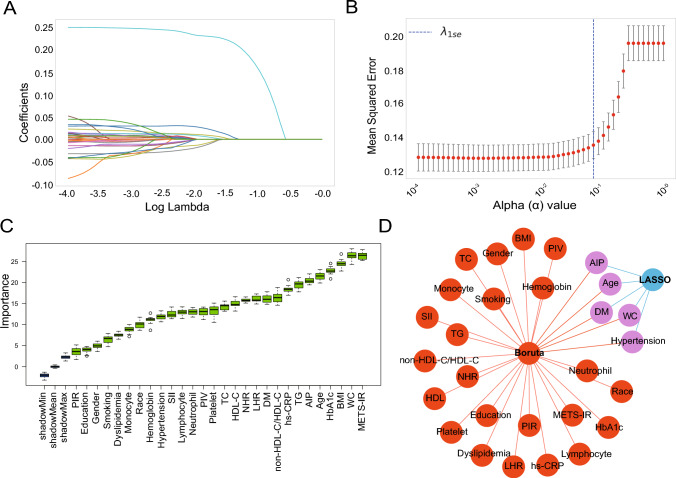
Variable selection using LASSO regression and Boruta algorithm. (**A**) The optimal parameter (λ) selection in LASSO is shown, with log(λ) plotted along the horizontal axis and the corresponding regression coefficients along the vertical axis; (**B**) The process of selecting the optimal λ value, where Alpha(α) value is represented on the horizontal axis, and the mean squared error (MSE) is displayed on the vertical axis; (**C**) Feature selection by Boruta; (**D**) Intersection of features selected by Boruta and LASSO. PIR, poverty income ratio; SII, systemic immune inflammation index; PIV, pan-immune-inflammation value; TC, total cholesterol; NHR, neutrophil-to-high-density lipoprotein cholesterol ratio; hs-CRP, hypersensitive-c-reactive-protein; DM, diabetes mellitus; LHR, lymphocyte-to-high-density lipoprotein ratio; HDL-C, high-density lipoprotein cholesterol; non-HDL-C/HDL-C, non-high-density lipoprotein cholesterol to high-density lipoprotein cholesterol; BMI, body mass index; WC, waist circumference

LASSO regression and Boruta algorithm were implemented to optimize variable selection. In the LASSO regression, variable selection was performed at lambda.1se, where the least number of variables and low MSE were observed. By taking the intersection of features selected by the two-step approach, five variables were identified from the training set, including age, WC, DM, hypertension, and AIP. Detailed process of variable selection is illustrated in Fig. 2.

### Model development and evaluation

**Fig. 3:**
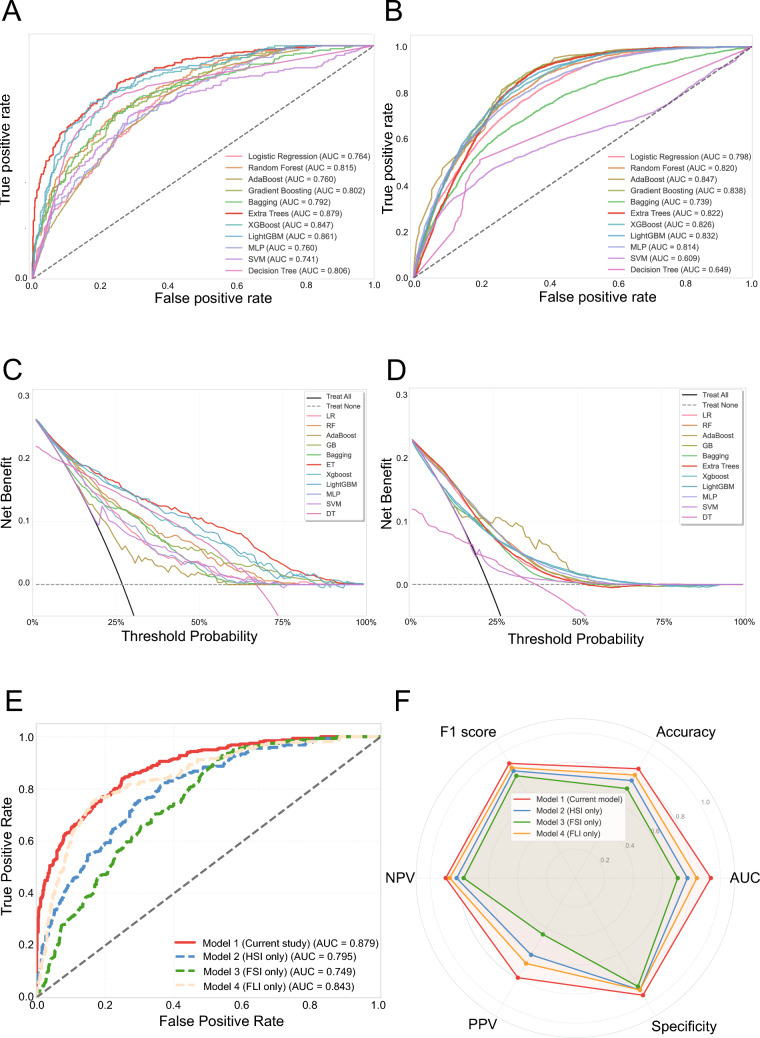
Performance of ML models for identifying MASLD. ROC curves for each ML-based model in the internal testing cohort (**A**) and external validation cohort (**B**). DCA curves for each model using eleven ML algorithms in the internal testing cohort (**C**) and external validation cohort (**D**). ROC and radar plot illustrating the predictive performance in the internal testing cohort (**E** and **F**). FLI, fatty liver index; FSI, Framingham steatosis index; HSI, hepatic steatosis index. ROC, receiver operating characteristic; ML, machine learning. DCA, decision curve analysis

**Fig. 4:**
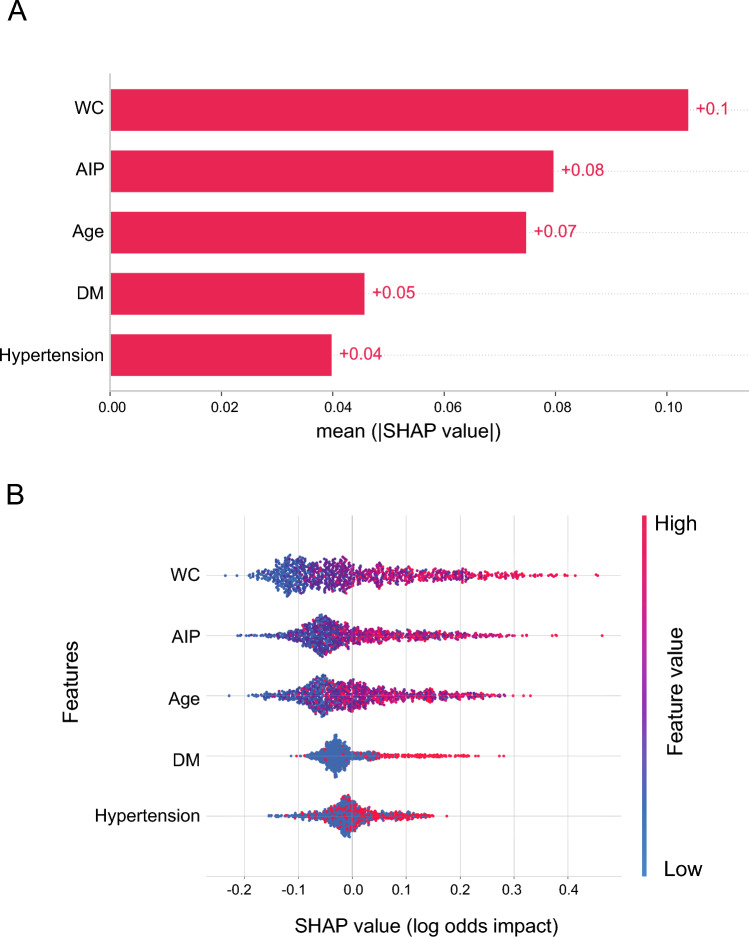
Variable importance rankings across different machine learning models explained by SHAP. (**A**) Importance bar chart. This plot evaluates the contribution of each feature to the model using mean SHAP values, displayed in descending order; (**B**) Beeswarm plot. The width of the range of horizontal bars can be interpreted as the impact on the model prediction that the wider its range, the larger its impact. The color of the horizontal bars represented the magnitude of predictors, which was coded in a gradient from blue (low) to red (high), shown as the color bar on the right-hand side. MASLD, metabolic dysfunction-associated steatotic liver disease. WC, waist circumference; AIP, atherogenic index of plasma

We utilized eleven ML algorithms based on the five selected variables to build classification models for identifying prevalent MASLD. Figure 3 presents the discriminative performance of the different algorithms. In internal testing set, the AUC of models ranged from 0.741 to 0.879 **(**Fig. 3A**)**. Interestingly, the ET model exhibited the greatest performance, achieving an AUC of 0.879 (95% CI 0.856–0.897), followed by LightGBM (AUC = 0.861, 95% CI 0.839–0.883), and XGBoost (AUC = 0.847, 95% CI 0.824–0.865). Further examination of the data in the internal testing group revealed that the ET model exhibited excellent accuracy, specificity, PPV, NPV, and F1 score of 0.834, 0.954, 0.801, 0.840, and 0.893, respectively. (Table S2 and Figure S2). In addition, the lowest Brier score for the ET model indicated the smallest error between model-estimated probabilities and observed MASLD status. These findings manifested that the ET model outperformed the ten other models across several performance metrics. What’s more, the Decision Curve Analysis (DCA) further supported its potential clinical utility for MASLD screening (Fig. 3C), showing the greatest net benefit across relevant threshold probabilities. This analysis illustrated that employing the ET model for risk prediction provided the maximal net benefits, underscoring its relevance in practical applications. To further examine the contribution of each feature, we analyzed the variable importance rankings produced by the ET algorithm **(**Fig. 4**)**. Our findings indicate that the WC emerged as the most significant predictor in the ET model.

### External validation of ML model

Model performance further validated by an external Asian cohort using data from 13944 subjects derived from the 2019–2021 KNHANES database. The analysis revealed that the ET model still exhibited a higher AUC value (Fig. 3B), consistent with the results of the internal testing set. Specifically, the ET Classifier algorithm showed an AUC of 0.822 (95% CI 0.815–0.829), which was slightly lower than the AdaBoost, GBoost, and LightGBM model’s AUC of 0.846 (95% CI 0.840–0.853), 0.838 (95% CI 0.831–0.845), 0.832 (95% CI 0.825–0.839), respectively. Although several algorithms achieved slightly higher AUCs in the external dataset, the ET model still retained acceptable discriminative performance in the external validation cohort. Visualization of the data was conducted to assess the predictive capabilities of the models within the external validation cohort, as depicted in Fig. 4B. Generally, the findings from the external validation set affirmed that the ET model exhibited excellent performance in the early prediction of MASLD. Importantly, pairwise AUC comparisons between the ET model and 10 other classification models confirmed the statistical significance of performance disparities, further validating the ET model’s robustness in predictive accuracy (**Table S3**).​

### Visualization by SHapley Additive exPlanations (SHAP)

To better understand the selected variables, we used the SHAP algorithm to characterize feature contributions within the ET model for MASLD identification. Figure 4A illustrates the five most influential features of the ET model and emphasizes their relevance. The features analyzed were organized in descending order according to their impact on the predicted outcomes, as indicated by the mean absolute values of SHAP. As shown in Fig. 4B shows the impact of each feature. Notably, WC and AIP were among the most influential features for distinguishing participants with MASLD from those without MASLD.

### Nonlinear relationships between predictors and MASLD

According to the feature selection results by LASSO regression and Boruta algorithm, we further explored the associations of continuous variables age, WC, and AIP ratio with MASLD (Figure S1). By applying restricted cubic splines, we examined the non-linear relationships between age, WC, AIP, and the modeled probability of prevalent MASLD after adjusting for confounding factors. Our results revealed significant overall and non-linear associations between the predictors and MASLD (*P* < 0.05 for both overall and non-linear effects). Notably, the estimated probability of prevalent MASLD increased markedly when age was above 23.02 years, AIP exceeded − 0.62, or WC surpassed 75.40 cm (Figure S1).

### Comparison of model predictive performance

We further compared the performance of ET model in this study with conventional hepatic steatosis related indices, such as FLI, FSI, and HSI [36, 37]. As shown in Fig. 3E and F, the performance of ET model was superior to that of conventional steatosis indices, including FLI, FSI, and HSI, in the internal testing cohort.

Lean MASLD represents a clinically important subgroup with potentially distinct metabolic characteristics [38, 39]. As shown in Figure S3, the selected five key predictors retained excellent discriminative performance for distinguishing lean from non-lean MASLD, with AUCs exceeding 0.90 in both the NHANES internal testing cohort and the KNHANES external validation cohort. The corresponding accuracy and specificity also remained high, further supporting the robustness of the model in this phenotype-based subgroup analysis.

## Discussion

This study developed an ML-based model for identifying prevalent MASLD by integrating demographic characteristics, clinical indices, and metabolic and immuno-inflammatory biomarkers. From all these variables, we identified five variables that are most relevant to the predictive performance of MASLD.

Currently, a standardized framework or consensus regarding features selection for prediction models is lacking, leading to uncertainty about the optimal number of features that should be incorporated. While a greater number of features may enrich the predictive capabilities of the model, an excess can hinder its clinical applicability. Our study employed a two-step feature selection, and found that five variables, including WC, DM, hypertension, AIP and age, are significantly associated with MASLD.

Numerous studies have proved the relationship between obesity, IR and MASLD [40, 41]. Similarly, in our study, three out of the five indicators selected by LASSO regression for model construction are related to obesity and IR, including WC, DM and AIP. The WC is a sign of obesity, while HDL-C is a quantifiable and monitorable markers of non-insulin-based IR [42, 43]. In obesity, adipocyte hypertrophy causes tissue hypoxia and mechanical stress, activating immune cells and promoting adipose tissue insulin resistance [41, 44]. In terms of IR state, the liver’s glucose metabolism regulation function of insulin is impaired, but the ability to promote lipid synthesis is retained, resulting in excessive lipid accumulation in the liver [45]. This phenomenon of “selective IR” is considered to be the core mechanism of MASLD.

AIP is a novel marker that evaluates the risk of atherosclerosis and reflects the degree of lipid metabolism abnormalities and IR [46]. It not only heightens the risk of IR but also disrupts lipid metabolic processes. Song et al. examined association between cardiometabolic indices and onset of MASLD. Interestingly, their found a strong relationship between AIP and MASLD, particularly among young females [47]. Similarly, another study found that individuals with higher AIP levels exhibited obviously increased risk of MAFLD, with the risk increasing by 12.42-fold for each 1-SD increase in AIP [48].

Our study also found that age had strong discriminative value for prevalent MASLD. Richell et al. have shown that the effect of age on MASLD may be related to sex hormones [49]. In the liver, sex hormones play an important role in regulating various biochemical and metabolic processes, like lipid metabolism and detoxification [50]. Estrogen is believed to have a protective effect on MASLD, by playing a significant role in lipogenesis, as well as in maintaining glucose and cholesterol homeostasis, while also enhancing insulin sensitivity [51]. Therefore, older women seem to be more susceptible to MASLD than younger women because of decreased estrogen levels.

We used the selected five variables to develop ML-based classification models. It should be noted that SHAP-derived feature importance reflects the contribution of variables to cross-sectional model discrimination rather than temporal or causal precedence. Among the eleven algorithms, the ET model achieved the highest AUC in the internal testing cohort and maintained good discriminative ability in the external validation cohort. In several prior studies [52, 53], the validation or testing set was used as the main dataset for identifying the optimal model, thereby demonstrating its efficacy and relative advantages across different contexts beyond the training data. Therefore, Fig. 2 only displays model’s AUC value in the internal testing set and external validation set, without including the training set. The ET classifier algorithm, as an ensemble learning method, exhibits significant advantages in predictive analytics. This approach leverages decision trees by employing multiple instances of them, hence the term "extra," as it builds on conventional tree-based methods to enhance predictive accuracy while mitigating overfitting [54]. Our final ET model represents a straightforward and user-friendly ML prediction tool that can facilitate clinical decision-making for the general population. In addition, we also compared the ET model’s performance with common hepatic steatosis indices. The findings revealed that this model was superior to conventional steatosis indices in the internal testing cohort.

While our research demonstrated meaningful progress in MASLD prediction, certain limitations must be recognized alongside opportunities for enhancement. First, model development was based on the NHANES database, with MASLD diagnosed using transient elastography (FibroScan®) in the training and internal validation cohorts. However, the KNHANES cohort lacked transient elastography data as well as other imaging or pathological data for MASLD diagnosis. Therefore, we used the HSI, a non-invasive screening tool developed in a Korean population and widely applied in epidemiological studies for assessing hepatic steatosis with reasonable accuracy [37, 55]. This methodological difference may explain the slightly lower performance of ET model compared to other algorithms in the external validation cohort. We also acknowledge that vibration-controlled transient elastography (VCTE) with CAP is a pragmatic non-invasive tool for steatosis assessment in population-based studies rather than a true gold standard. Compared with MRI-PDFF (proton density fat fraction) or liver biopsy [56], VCTE may be less accurate and may have higher technical failure or unreliability in individuals with obesity, which could have introduced some degree of outcome misclassification in our study. Second, because both NHANES and KNHANES are cross-sectional datasets, the selected variables should be interpreted as correlates of prevalent MASLD rather than predictors of future onset, and the study cannot support causal inference or longitudinal risk stratification. Third, only patients with complete records were included in the study, and selection bias is hard to avoid. Fourth, our study did not differentiate the subtypes or severity levels of MASLD, nor did it assess whether MASLD patients had liver fibrosis, which may have compromised the rigor of the model. In the future, incorporating MASLD subtyping data may help establish models that distinguish MASLD subtypes rather than predict their future occurrence. Fifth, considering the clinical practicality and convenience of the model, we included only conventional demographic and clinical indicators, while newer molecular markers such as CXCL10 and CK-18 were not incorporated into the study [57]. Finally, the use of lipid-lowering and antidiabetic medications may have modified routine laboratory biomarkers and derived metabolic indices, introducing residual confounding that could have influenced the observed associations and model performance. Despite these limitations, our predictive model remains a practical and well-performing diagnostic tool for assessing MASLD risk.

## Conclusion

In conclusion, ML models may serve as useful tools for screening and identifying prevalent MASLD in adults. Among these variables, age, WC, DM, hypertension, and AIP were the most informative features associated with prevalent MASLD. The use of demographic characteristics and routine laboratory biomarkers, combined with ML algorithms, may improve MASLD case identification.

## Supplementary Information

Below is the link to the electronic supplementary material.

Supplementary file1 (PDF 799 KB)

## Data Availability

No datasets were generated or analysed during the current study.

## Abbreviations

MASLD: Metabolic dysfunction-associated steatotic liver disease
NHANES: National Health and Nutrition Examination Survey
ML: Machine learning
LASSO: Least absolute shrinkage and selection operator
FLI: Fatty liver index
HSI: Hepatic steatosis index
FSI: Framingham steatosis index
BMI: Body mass index
WC: Waist circumference
IR: Insulin resistance
HDL-C: High-density lipoprotein cholesterol
LDL-C: Low-density lipoprotein cholesterol
TG: Triglycerides
TC: Total cholesterol
LHR: Lymphocyte-to-high-density lipoprotein ratio
NHR: Neutrophil-to-high-density lipoprotein cholesterol ratio
SII: Systemic immune inflammation index
PIV: Pan-immune-inflammation value
AIP: Atherogenic index of plasma
SVM: Support vector machine
MLP: Multilayer perceptron
LR: Logistic regression
DT: Decision trees
RF: Random forest
ET: Extremely randomized trees
Bagging: Bootstrap aggregating
AdaBoost: Adaptive boosting
GBoost: Gradient boosting
XGBoost: Extreme gradient boosting
LightGBM: Light gradient boosting machine
SHAP: SHapley Additive exPlanations
AUC: Area under the curve
ROC: Receiver operating characteristic
CAP: Controlled attenuation parameter
LSM: Liver stiffness measurement
