# Supplementary material for: Development and validation of an explainable machine learning model using routine laboratory biomarkers for identifying prevalent MASLD: Evidence from two observational studies
Source: Clin Exp Med. 2026 May 15;26(1):257. doi: 10.1007/s10238-026-02163-x (PMC13346284; doi:10.1007/s10238-026-02163-x)
Supplement: Supplementary file 1 — Supplementary file1 (PDF 799 KB) [file 10238_2026_2163_MOESM1_ESM.pdf]

Supplementary

Development and validation of an explainable machine learning model using routine laboratory biomarkers for identifying prevalent MASLD: Evidence from two observational studies

Contents

目录

Supplementary method 2

Figure S1 3

Figure S2 4

Figure S3 5

Table S1 6

Table S2 8

Table S3 9

Supplementary method

The current study external cohort was analyzing data from the 2019–2021 Korean National Health and Nutrition Examination Survey (KNHANES). The KNHANES is a nationwide representative survey conducted by the Korea Disease Control and Prevention Agency to assess the health and nutritional status of Korean citizens. This study was a secondary analysis of publicly available, de-identified data from the KNHANES, using only disclosed variables. Therefore, additional ethical approval was not required. Further details regarding the survey design and data collection are available on the KNHANES website.

Among the 22,559 participants who participated in the KNHANES from 2019 to 2021, we excluded participants with the following characteristics: (1) age less than 20 years (N = 4048); (2) heavy alcohol drinker (N = 1691); (3) hepatitis B or C viral carrier (N = 980); and (4) pregnancy and insufficient data to calculate hepatic steatosis (N = 16,251). Therefore, we finally selected 13,944 participants for this study.

In KNHANES cohort, metabolic dysfunction associated steatotic liver disease (MASLD) was defined as the presence of hepatic steatosis along with one or more of the following cardiometabolic risk factors1,2: (1) overweight or obesity or abdominal obesity (body mass index: ≥25 kg/m2 or waist circumference: ≥90 cm (men) or ≥80 cm (women)), (2) impaired fasting glucose (fasting glucose ≥126 mg/dl), (3) diagnosis of diabetes mellitus or HbA1c ≥6.5%3, (4) high blood pressure ([BP] ≥140/90 mmHg, high total cholesterol (≥20 mg/dl), or the use of antihypertensive medications, and (5) low high-density lipoprotein (<40 mg/dL in men or < 50 ml/dL in women) or the use of lipid-lowering drugs. Hepatic steatosis was evaluated using the hepatic steatosis index (HSI), which was developed based on the Korean population and is a widely used non-invasive screening tool for evaluating hepatic steatosis in epidemiological studies with a reasonable degree of accuracy4,5. The HSI was calculated based on the alanine aminotransferase to aspartate aminotransferase ratio (ALT/AST ratio), presence of DM, and sex (8 × ALT/AST ratio + 2 [if DM] + 2 [if female]), with values ≥ 36 indicating the presence of hepatic steatosis. In the KNHANES cohort, MASLD was diagnosed using the hepatic steatosis index (HSI ≥ 36) combined with metabolic criteria.

References:

1 Rinella, M. E. *et al.* A multisociety Delphi consensus statement on new fatty liver disease nomenclature. *Hepatology* **78**, 1966-1986, doi:10.1097/HEP.0000000000000520 (2023).

2 Han, E. *et al.* Impact of physical activities in metabolic dysfunction associated steatotic liver disease, sarcopenia, and cardiovascular disease. *Diabetes Res Clin Pract* **224**, 112209, doi:10.1016/j.diabres.2025.112209 (2025).

3 Moon, J. S. *et al.* 2023 Clinical Practice Guidelines for Diabetes Management in Korea: Full Version Recommendation of the Korean Diabetes Association. *Diabetes Metab J* **48**, 546-708, doi:10.4093/dmj.2024.0249 (2024).

4 Lee, J.-H. *et al.* Hepatic steatosis index: a simple screening tool reflecting nonalcoholic fatty liver disease. *Dig Liver Dis* **42**, 503-508, doi:10.1016/j.dld.2009.08.002 (2010).

5 Baek, S. U., Won, J. U., Lee, Y. M. & Yoon, J. H. Association between long working hours and metabolic dysfunction-associated steatotic liver disease: a nationwide population-based study in Korea. *Public Health* **232**, 188-194, doi:10.1016/j.puhe.2024.04.034 (2024).

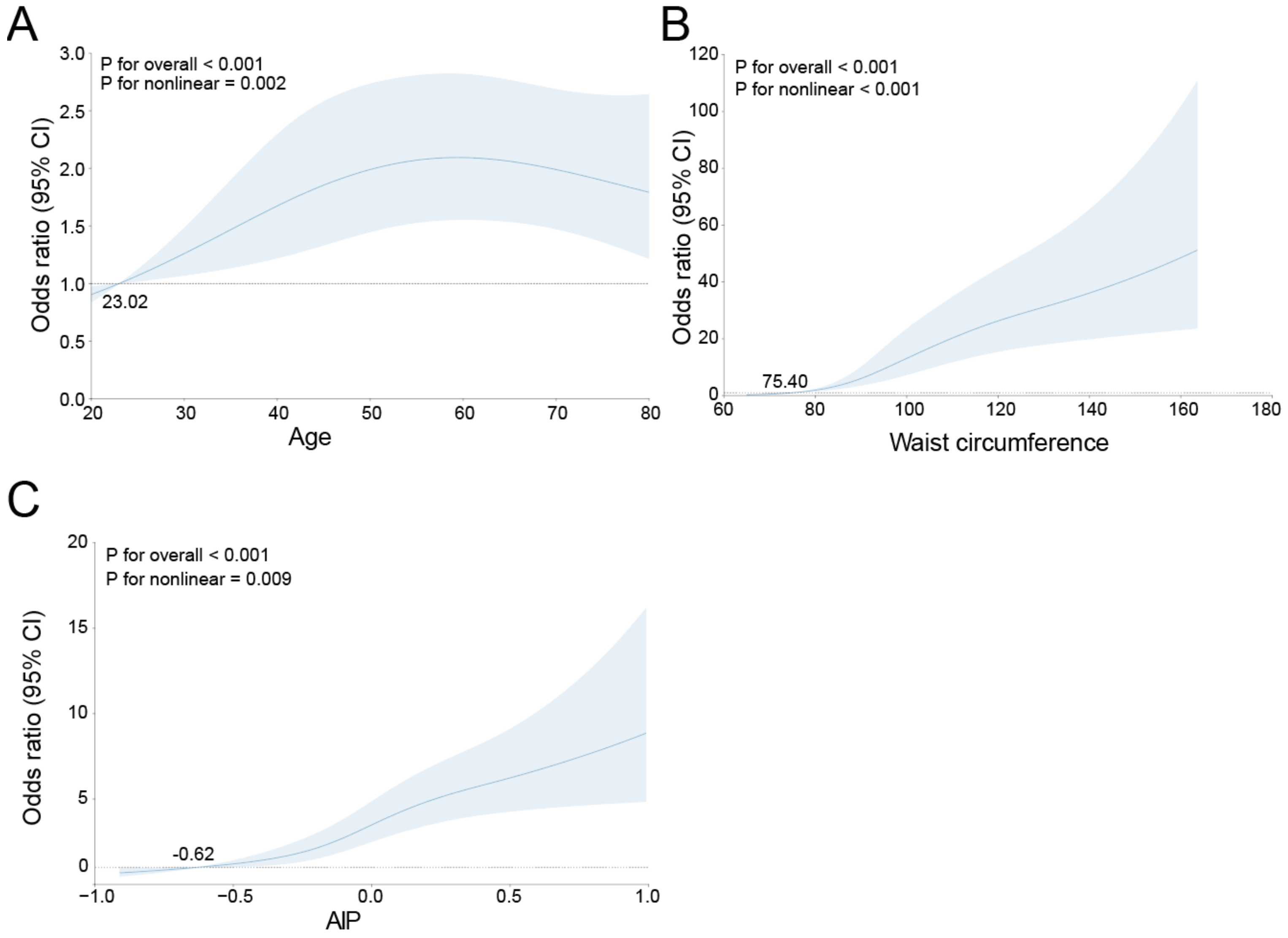

Figure S1 Restricted cubic spline plots for different continuous variables. These plots illustrate the nonlinear relationships between each continuous variable and MASLD. The specific continuous variables include (A) Age; (B) Waist circumference; (C) AIP. Each variable's overall and nonlinear relationships are accompanied by P-values, indicating the significance and nonlinearity of the associations. AIP, atherogenic index of plasma.

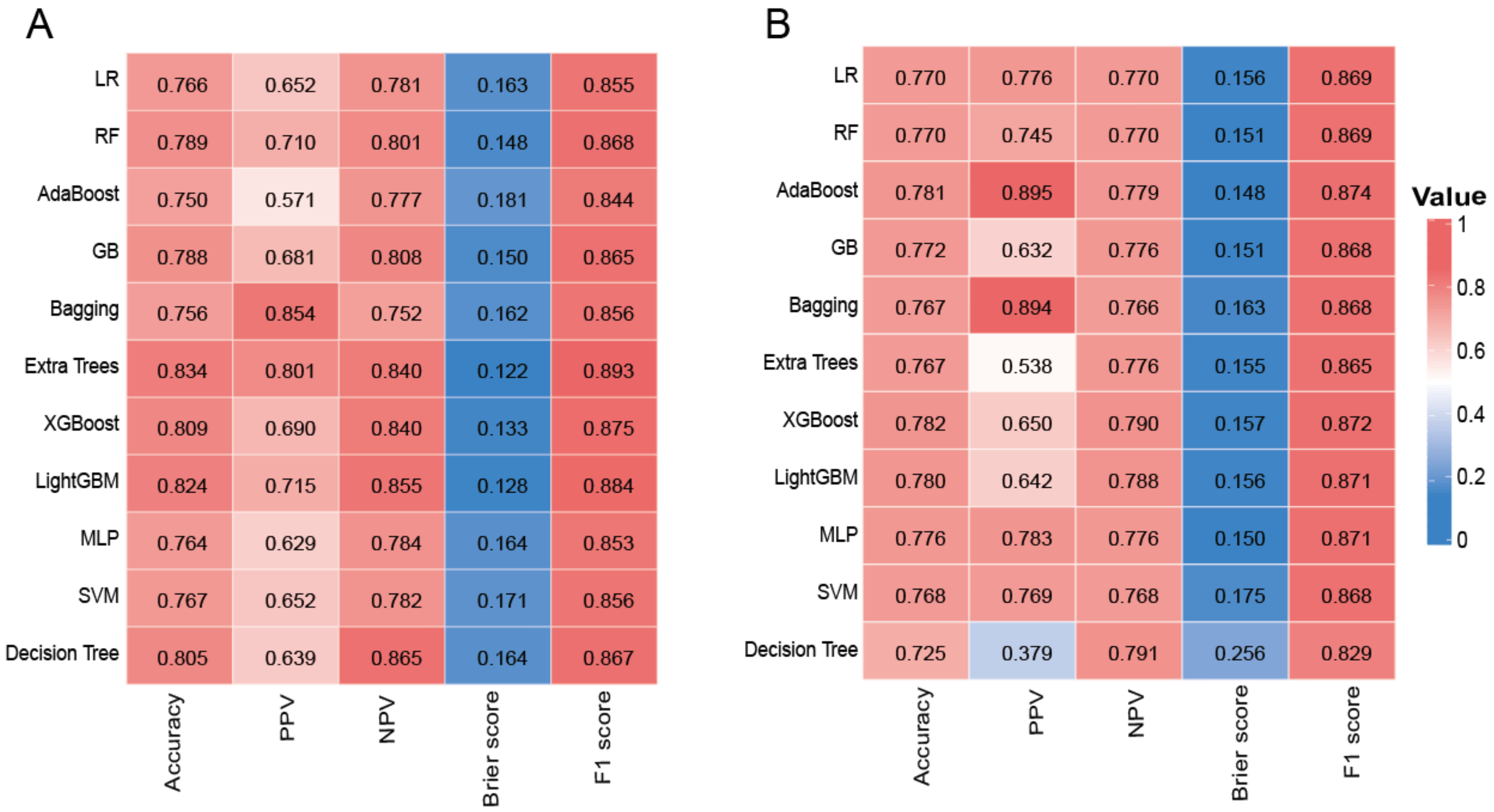

Figure S2 Heatmap of the evaluation metrics in eleven machine learning models. Color scale represents the value of different evaluation metrics (red: value between 0.5-1.0; blue: value between 0-0.5). (A) The internal testing cohort; (B) The external validation cohort. LR, logistic regression; RF, random forest; GB, gradient boosting; SVM. support vector machine; MLP, multilayer perceptron; AdaBoost, adaptive boosting; XGBoost, extreme gradient boosting; LightGBM, light gradient boosting machine; Bagging, bootstrap aggregating; PPV, positive predictive value; NPV, negative predictive value.

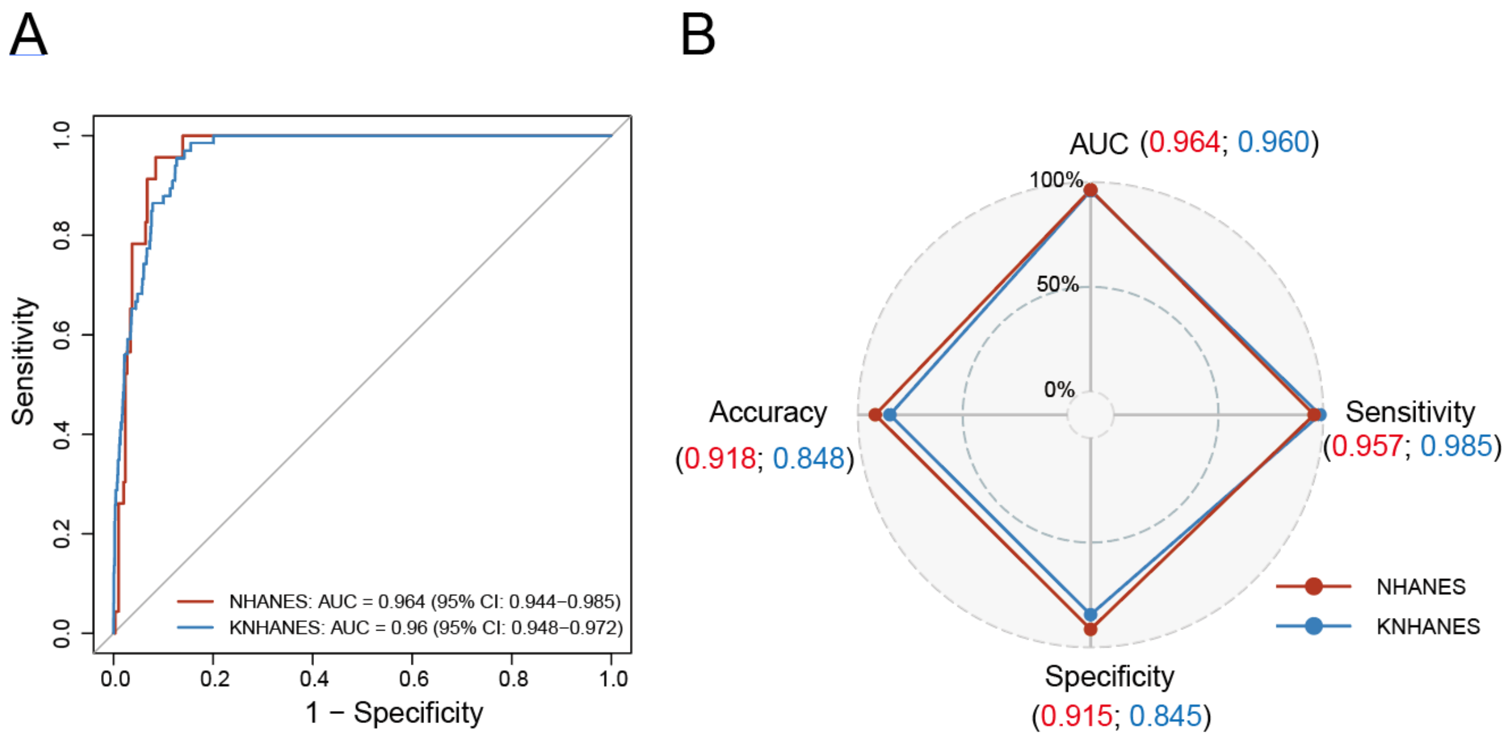

Figure S3 Performance of the clinical model for discriminating lean from non-lean MASLD in the NHANES internal testing cohort and the KNHANES external validation cohort. (A) Receiver operating characteristic curves. (B) Radar plot summarizing the area under the curve, sensitivity, specificity, and accuracy.

Table S1 Comparison of characteristics between MASLD and non-MASLD participants.

Characteristics	Training set			Internal testing set		
	Non-MASLD (N=2020)	MASLD (N=740)	*P*-value	Non-MASLD (N=998)	MASLD (N=334)	*P*-value
**Demographic characteristics**						
Age	47.0 (33.00, 62.00)	57.0 (44.00, 68.00)	<0.001	48.0 (33.00, 61.00)	58.0 (45.00, 67.00)	<0.001
Gender, n (%)			0.009			0.001
Female	1079 (53.4%)	353 (47.7%)		484 (55.9%)	143 (45.0%)	
Male	941 (46.6%)	387 (52.3%)		382 (44.1%)	175 (55.0%)	
Race, n (%)			0.001			0.275
Mexican American	255 (12.6%)	111 (14.3%)		115 (13.3%)	48 (15.1%)	
Non-Hispanic White	691 (34.2%)	303 (40.9%)		300 (34.6%)	126 (39.6%)	
Non-Hispanic Black	614 (25.4%)	141 (19.1%)		226 (26.1%)	67 (21.1%)	
Other Hispanic	201 (10.0%)	70 (9.5%)		84 (9.7%)	26 (8.2%)	
Other races	359 (17.8%)	122 (16.5%)		141 (16.3%)	51 (16.0%)	
PIR, n (%)			0.745			0.056
＜1	315 (17.4%)	124 (16.8%)		172 (19.9%)	47 (14.8%)	
≥1	1669 (82.6%)	616 (83.2%)		694 (80.1%)	271 (85.2%)	
Education, n (%)			0.356			0.717
Less than high school	355 (17.4%)	124 (16.8%)		154 (17.8%)	53 (16.7%)	
High school graduate +	1669 (82.6%)	616 (83.2%)		712 (82.2%)	265 (83.3%)	
BMI (kg/m^2)	27.6 (23.80, 32.50)	32.2 (28.37, 37.30)	<0.001	27.4 (24.00, 32.30)	32.2 (28.22, 37.40)	<0.001
Waist circumference (cm)	96.4 (85.50, 108.10)	110.3 (100.30, 120.60)	<0.001	96.0 (85.32, 107.88)	108.8 (99.05, 120.80)	<0.001
Smoking, n (%)			0.653			0.025
No	1180 (58.4%)	440 (59.5%)		494 (57.0%)	205 (64.5%)	
Yes	840 (41.6%)	300 (40.5%)		372 (43.0%)	113 (35.5%)	
**Metabolic syndrome**						
DM, n (%)			<0.001			<0.001
No	1661 (82.2%)	421 (56.9%)		736 (85.0%)	184 (57.9%)	
Yes	359 (17.8%)	319 (43.1%)		130 (15.0%)	134 (42.1%)	
Hypertension, n (%)			<0.001			<0.001
No	1236 (61.2%)	286 (38.6%)		528 (61.0%)	129 (40.6%)	
Yes	784 (38.8%)	454 (61.4%)		338 (39.0%)	189 (59.4%)	
Dyslipidemia, n (%)			<0.001			<0.001
No	696 (34.5%)	113 (15.3%)		294 (33.3%)	54 (17.0%)	
Yes	1324 (65.5%)	627 (84.7%)		572 (66.1%)	264 (83.0%)	
METS-IR	40.2 (32.94, 49.16)	50.2 (43.33, 59.82)	<0.001	39.6 (33.22, 48.26)	50.0 (43.04, 59.15)	<0.001
**Laboratory test**						
Lymphocyte (10^9/L)	2.0 (1.60, 2.30)	2.0 (1.70, 2.50)	0.001	2.0 (1.60, 2.40)	2.0 (1.60, 2.48)	0.539
Monocyte (10^9/L)	0.5 (0.40, 0.60)	0.5 (0.40, 0.60)	<0.001	0.5 (0.40, 0.60)	0.5 (0.40, 0.70)	0.038
Neutrophil (10^9/L)	3.6 (2.80, 4.60)	4.0 (3.20, 5.00)	<0.001	3.6 (2.80, 4.60)	4.1 (3.10, 5.20)	<0.001
Platelet (10^9/L)	233.0 (196.00, 277.00)	234.5 (198.00, 270.00)	0.748	236.0 (196.00, 275.50)	237.5 (200.25, 280.75)	0.149
Hemoglobin (g/dL)	14.1 (13.20, 15.10)	14.3 (13.30, 15.38)	0.059	14.0 (13.00, 15.00)	14.1 (13.20, 15.20)	0.111

HbA1c (%)	5.5 (5.30, 5.90)	5.9 (5.50, 6.70)	<0.001	5.5 (5.30, 5.80)	5.8 (5.50, 6.68)	<0.001
HDL-C (mg/dL)	52.0 (43.00, 62.00)	44.0 (39.00, 53.00)	<0.001	53.0 (44.00, 63.00)	46.0 (39.00, 54.00)	<0.001
hs-CRP (mg/L)	1.7 (0.75, 4.01)	3.0 (1.37, 6.00)	<0.001	1.7 (0.73, 4.05)	2.5 (1.18, 5.09)	<0.001
TC (mg/dL)	180.0 (156.00, 209.00)	180.0 (155.00, 207.00)	0.866	180.0 (156.00, 210.00)	183.0 (156.25, 212.50)	0.475
**Indices**						
NHR	2.6 (1.84, 3.79)	3.4 (2.55, 4.68)	<0.001	2.6 (1.81, 3.80)	3.5 (2.43, 4.65)	<0.001
LHR	1.5 (1.06, 1.94)	1.7 (1.31, 2.26)	<0.001	1.4 (1.07, 1.94)	1.7 (1.24, 2.25)	<0.001
AIP	-0.20 (-0.37, 0.07)	0.10 (-0.13, 0.24)	<0.001	-0.2 (-0.38, 0.03)	0.0 (-0.17, 0.22)	<0.001
non-HDL-C/HDL-C ratio	2.4 (1.76, 3.24)	2.9 (2.26, 3.76)	<0.001	2.4 (1.73, 3.19)	2.9 (2.17, 3.75)	<0.001
SII	428.9 (305.51, 604.87)	445.5 (320.81, 639.37)	0.008	432.4 (306.22, 602.12)	469.8 (324.72, 670.62)	0.005
PIV	211.4 (137.70, 334.83)	245.5 (157.21, 365.05)	<0.001	216.8 (141.81, 340.44)	256.8 (151.69, 419.59)	0.001
CAP (dB/m)	241.0 (209.00, 275.00)	323.0 (298.00, 350.50)	<0.001	241.0 (209.00, 271.00)	320.0 (297.00, 348.00)	<0.001
LSM (kPa)	4.8 (4.00, 5.90)	5.6 (4.50, 7.10)	<0.001	4.8 (3.90, 5.90)	5.6 (4.60, 7.90)	<0.001
HSI	36.8 (31.72, 42.92)	43.4 (38.48, 48.70)	<0.001	36.2 (31.72, 42.14)	43.7 (38.18, 48.89)	<0.001
FLI	42.5 (13.95, 78.50)	82.5 (59.62, 94.58)	<0.001	41.6 (13.51, 77.04)	80.0 (53.47, 95.48)	<0.001
FSI	-1.6 (-2.71, 0.52)	-0.2 (-1.06, 0.90)	<0.001	-1.7 (-2.70, -0.61)	-0.3 (-1.12, 0.92)	<0.001

Note: Continuous variables are presented as median (IQR). Categorical variables are presented as n (%).

Abbreviations: PIR, poverty income ratio; BMI, body mass index; DM, diabetes mellitus; METS-IR, metabolic score for insulin resistance; HDL-C, high-density lipoprotein cholsterol; hs-CRP, hypersensitive-c-reactive-protein; TG, triglycerides; TC, total cholesterol; NHR, neutrophil-to-high-density lipoprotein cholesterol ratio; LHR, lymphocyte-to-high-density lipoprotein ratio; AIP, atherogenic index of plasma; non-HDL-C/HDL-C, non-high-density lipoprotein cholesterol to high-density lipoprotein cholesterol ratio; SII, systemic immune inflammation index; PIV, pan-immune-inflammation value; CAP, controlled attenuation parameter; LSM, liver stiffness measurement; FLI, fatty liver index; FSI, Framingham steatosis index; HIS, hepatic steatosis index; MASLD, metabolic dysfunction associated steatotic liver disease.

Table S2 Performance of various machine learning models for predicting MASLD across internal testing and external validation cohorts.

Model	Accuracy (95% CI)	Recall (95% CI)	Specificity (95% CI)	PPV (95% CI)	NPV (95% CI)	Brier score (95% CI)	F1 score (95% CI)
**Internal testing cohort**							
LR	0.766 (0.744, 0.790)	0.277 (0.223, 0.333)	0.946 (0.932, 0.960)	0.652 (0.571, 0.732)	0.781 (0.754, 0.805)	0.163 (0.151, 0.176)	0.855 (0.840, 0.871)
RF	0.789 (0.766, 0.814)	0.362 (0.308, 0.424)	0.946 (0.930, 0.961)	0.710 (0.648, 0.786)	0.801 (0.776, 0.824)	0.148 (0.138, 0.160)	0.868 (0.851, 0.884)
AdaBoost	0.750 (0.727, 0.771)	0.277 (0.227, 0.329)	0.924 (0.907, 0.940)	0.571 (0.497, 0.654)	0.777 (0.756, 0.798)	0.181 (0.176, 0.187)	0.844 (0.827, 0.858)
GBoost	0.788 (0.760, 0.807)	0.396 (0.342, 0.452)	0.932 (0.915, 0.946)	0.681 (0.616, 0.736)	0.808 (0.783, 0.829)	0.150 (0.140, 0.163)	0.865 (0.846, 0.879)
Bagging	0.756 (0.732, 0.779)	0.110 (0.072, 0.144)	0.993 (0.987, 0.998)	0.854 (0.735, 0.951)	0.752 (0.726, 0.775)	0.162 (0.153, 0.173)	0.856 (0.838, 0.871)
ET	0.834 (0.811, 0.854)	0.506 (0.442, 0.567)	0.954 (0.940, 0.966)	0.801 (0.758, 0.853)	0.840 (0.813, 0.857)	0.122 (0.113, 0.132)	0.893 (0.877, 0.907)
XGBoost	0.809 (0.787, 0.827)	0.525 (0.466, 0.578)	0.913 (0.893, 0.931)	0.690 (0.633, 0.747)	0.840 (0.816, 0.861)	0.133 (0.124, 0.145)	0.875 (0.858, 0.888)
LightGBM	0.824 (0.805, 0.845)	0.575 (0.512, 0.634)	0.916 (0.902, 0.932)	0.715 (0.669, 0.764)	0.855 (0.831, 0.875)	0.128 (0.114, 0.139)	0.884 (0.869, 0.899)
MLP	0.764 (0.742, 0.791)	0.299 (0.252, 0.357)	0.935 (0.920, 0.949)	0.629 (0.565, 0.707)	0.784 (0.760, 0.810)	0.164 (0.152, 0.176)	0.853 (0.835, 0.870)
SVM	0.767 (0.741, 0.790)	0.283 (0.236, 0.340)	0.945 (0.930, 0.959)	0.652 (0.578, 0.734)	0.782 (0.755, 0.807)	0.171 (0.160, 0.183)	0.856 (0.838, 0.871)
DT	0.805 (0.779, 0.826)	0.629 (0.568, 0.672)	0.870 (0.848, 0.891)	0.639 (0.593, 0.689)	0.865 (0.836, 0.884)	0.164 (0.147, 0.186)	0.867 (0.847, 0.885)
**External validation cohort**							
LR	0.770 (0.763, 0.777)	0.036 (0.029, 0.043)	0.997 (0.996, 0.998)	0.776 (0.710, 0.842)	0.770 (0.763, 0.777)	0.156 (0.152, 0.160)	0.869 (0.864, 0.873)
RF	0.770 (0.763, 0.777)	0.040 (0.033, 0.046)	0.996 (0.995, 0.997)	0.745 (0.677, 0.810)	0.770 (0.763, 0.778)	0.151 (0.147, 0.155)	0.869 (0.864, 0.873)
AdaBoost	0.781 (0.774, 0.788)	0.083 (0.073, 0.092)	0.997 (0.996, 0.998)	0.895 (0.860, 0.929)	0.779 (0.772, 0.785)	0.148 (0.146, 0.150)	0.874 (0.870, 0.879)
GBoost	0.772 (0.765, 0.779)	0.079 (0.070, 0.089)	0.986 (0.984, 0.988)	0.632 (0.582, 0.678)	0.776 (0.769, 0.783)	0.151 (0.147, 0.155)	0.868 (0.864, 0.873)
Bagging	0.767 (0.760, 0.774)	0.014 (0.010, 0.018)	1.000 (0.999, 1.000)	0.894 (0.796, 0.973)	0.766 (0.759, 0.774)	0.163 (0.159, 0.166)	0.868 (0.863, 0.872)
ET	0.767 (0.760, 0.774)	0.088 (0.079, 0.098)	0.977 (0.974, 0.980)	0.538 (0.493, 0.581)	0.776 (0.769, 0.783)	0.155 (0.151, 0.159)	0.865 (0.860, 0.869)
XGBoost	0.782 (0.775, 0.789)	0.161 (0.148, 0.174)	0.973 (0.970, 0.977)	0.650 (0.616, 0.684)	0.790 (0.783, 0.797)	0.157 (0.152, 0.162)	0.872 (0.867, 0.877)
LightGBM	0.780 (0.773, 0.787)	0.151 (0.139, 0.164)	0.974 (0.971, 0.977)	0.642 (0.605, 0.678)	0.788 (0.781, 0.795)	0.156 (0.151, 0.161)	0.871 (0.867, 0.876)
MLP	0.776 (0.768, 0.783)	0.068 (0.060, 0.077)	0.994 (0.993, 0.996)	0.783 (0.734, 0.832)	0.776 (0.768, 0.783)	0.150 (0.146, 0.154)	0.871 (0.867, 0.876)
SVM	0.768 (0.761, 0.775)	0.027 (0.021, 0.032)	0.998 (0.997, 0.998)	0.769 (0.687, 0.844)	0.768 (0.761, 0.776)	0.175 (0.171, 0.179)	0.868 (0.864, 0.873)
DT	0.725 (0.718, 0.733)	0.257 (0.243, 0.271)	0.870 (0.863, 0.877)	0.379 (0.358, 0.399)	0.791 (0.784, 0.799)	0.256 (0.249, 0.263)	0.829 (0.823, 0.834)

Abbreviations: LR, logistic regression; RF, random forest; AdaBoost, adaptive boosting; GBoost, gradient boosting; Bagging, bootstrap aggregating; ET, extremely randomized trees; XGBoost, extreme gradient boosting; LightGBM, light gradient boosting machine; MLP, multilayer perceptron; SVM, support vector machine; DT, decision trees; PPV, positive predictive value; NPV, negative predictive value.

Table S3 Pairwise comparison of ROC curves for classification models using DeLong's test in the internal testing cohort and external validation cohort

Compared Model	ET AUC	Model AUC	Delta-AUC	Z-score	*P*-value
**Internal testing cohort**					
SVM	0.879	0.741	0.138	10.471	<0.001
DT	0.879	0.806	0.073	5.718	<0.001
AdaBoost	0.879	0.760	0.119	11.372	<0.001
MLP	0.879	0.760	0.118	11.556	<0.001
LR	0.879	0.764	0.115	11.428	<0.001
GBoost	0.879	0.802	0.077	9.303	<0.001
LightGBM	0.879	0.861	0.018	2.731	0.006
XGBoost	0.879	0.847	0.032	4.436	<0.001
RF	0.879	0.815	0.064	9.084	<0.001
Bagging	0.879	0.792	0.087	7.329	<0.001
**External validation cohort**					
SVM	0.822	0.609	0.213	32.817	<0.001
DT	0.822	0.649	0.173	36.394	<0.001
AdaBoost	0.822	0.846	-0.024	-8.239	<0.001
MLP	0.822	0.814	0.008	2.894	0.004
LR	0.822	0.798	0.025	8.706	<0.001
GBoost	0.822	0.838	-0.015	-6.628	<0.001
LightGBM	0.822	0.832	-0.010	-4.192	<0.001
XGBoost	0.822	0.825	-0.003	-1.228	0.219
RF	0.822	0.820	0.002	1.238	0.216
Bagging	0.822	0.739	0.083	18.208	<0.001

Abbreviations: MLP, multilayer perceptron; XGBoost, extreme gradient boosting; LR, logistic regression; LightGBM, light gradient boosting machine; Bagging, bootstrap aggregating; SVM, support vector machine; DT, decision trees; ET, extremely randomized trees; AdaBoost, adaptive boosting; RF, random forest; GBoost, gradient boosting; AUC, area under the curve.
